# Modelling the Temperature Dependent Biaxial Response of Poly(ether-ether-ketone) Above and Below the Glass Transition for Thermoforming Applications

**DOI:** 10.3390/polym11061042

**Published:** 2019-06-12

**Authors:** Josh A. Turner, Gary H. Menary, Peter J. Martin, Shiyong Yan

**Affiliations:** School of Mechanical and Aerospace Engineering, Queen’s University, Belfast BT9 5AH, UK; g.menary@qub.ac.uk (G.H.M.); p.j.martin@qub.ac.uk (P.J.M.); s.yan@qub.ac.uk (S.Y.)

**Keywords:** PEEK, constitutive model, anisotropy, simulation, thermoforming

## Abstract

Desire to accurately predict the deformation behaviour throughout industrial forming processes, such as thermoforming and stretch blow moulding, has led to the development of mathematical models of material behaviour, with the ultimate aim of embedding into forming simulations enabling process and product optimization. Through the use of modern material characterisation techniques, biaxial data obtained at conditions comparable to the thermoforming process was used to calibrate the Buckley material model to the observed non-linear viscoelastic stress/strain behaviour. The material model was modified to account for the inherent anisotropy observed between the principal directions through the inclusion of a Holazapfel–Gasser–Ogden hyperelastic element. Variations in the post-yield drop in stress values associated with deformation rate and specimen temperature below the glass transition were observable, and facilitated in the modified model through time-temperature superposition creating a linear relationship capable of accurately modelling this change in yield stress behaviour. The modelling of the region of observed flow stress noted when above the glass transition temperature was also facilitated through adoption of the same principal. Comparison of the material model prediction was in excellent agreement with experiments at strain rates and temperatures of 1–16 s^−1^ and 130–155 °C respectively, for equal-biaxial mode of deformation. Temperature dependency of the material model was well replicated with across the broad temperature range in principal directions, at the reference strain rate of 1 s^−1^. When concerning larger rates of deformation, minimum and maximum average error levels of 6.20% and 10.77% were noted. The formulation, and appropriate characterization, of the modified Buckley material model allows for a stable basis in which future implementation into representative forming simulations of poly-aryl-ether-ketones, poly(ether-ether-ketone) (PEEK) and many other post-yield anisotropic polymers.

## 1. Introduction

A semi-crystalline polymer belonging to the family of poly-aryl-ether-ketones, poly(ether-ether-ketone) (PEEK) is an emerging polymer gaining popularity for implementation into a wide range of applications in bulk form and as a matrix material for Short Fibre Reinforced Composites (SFRC) as a result of its superior mechanical qualities combined with high thermal stability and chemical resistance. The anticipated suitability of PEEK for the application of thin walled, thermoformed products is governed by its characteristically large stiffness to mass ratio (2.571 MNm/kg), or specific stiffness, when compared to similar thermoplastics in this field such as poly(ethylene terephthalate) (PET) and polypropylene (PP)—exhibiting stiffness to mass ratios of 1.206 and 1.935 MNm/kg respectively.

The industrial practice of thermoforming is widely used for the large-scale manufacture of lightweight, thin-walled polymeric parts with the advantage of producing repeatable, complex products, along with relatively cheap production costs. During the thermoforming process, the thermoplastic material is subject to a range of dynamic multi-axial deformation at strain rates of 1–10 s^−1^ and higher whilst processing the polymer in its rubber-like state above and below its characteristic glass transition temperature (T_g_) [1]. In many applications the relationship between the resultant deformation of the polymeric sheet is generally unknown, leading to local inhomogeneity in final part thicknesses. Trial and error approaches in the hopes of optimising the formed part is often time consuming, causing authors to take a more systematic methodology. This is predominantly achieved through the use of a mathematical material model to represent the deformation behaviour subject to characteristic loading experienced during forming processes. In order for this mathematical model to be accurate in its predictions of the mechanical behaviour of the polymeric material, characterisation at conditions comparable to those experienced during the forming process is encouraged [2,3,4]—allowing the constitutive model to be calibrated and then implemented into a process simulation [5,6,7] with the ultimate aim of optimising the forming conditions and/or the formed part.

The mechanical properties of PEEK have been widely investigated in previous work. Due to its many applications, authors have rigorously investigated the mechanical response of PEEK subject to various characterisation methods. An extensive review of these is presented by Rae et al. [8]. Mechanical characterisation of PEEK has been primarily focussed on uniaxial stretching [8,9,10,11,12], with authors detailing the strong temperature and deformation rate dependence of specimens. A lack of research concerning the deformation in more than one axis is evident, and has only been investigated recently by the authors [13]. In our previous study, both load-controlled and displacement-controlled testing of thin extruded PEEK specimens were conducted at conditions comparable to the thermoforming process, drawing similar parallels to the temperature and strain rate dependencies found during uniaxial tests as well as noting profound anisotropy when simultaneously stretching in two axes [13]. It is the data from this investigation which serves as the basis for modelling the constitutive biaxial deformation behaviour of PEEK, for the application of thermoforming.

Attempts to model the constitutive response of PEEK have only become apparent in the past decade, with mathematical models developed to quantify specific mechanical properties. El-Halabi et al. [14] characterised and modelled the yield stress of PEEK for scaffold cranial implants, through three point bending tests. The data was then used to calibrate homogenized material models and predict local areas of fracture within scaffold designs in finite element models. El-Qoubaa and Othman investigated the discernible influence of deformation rate on the uniaxial tensile and compressive yield stresses over a wide strain rate range [11,15] and temperature range spanning the glass transition [16]. In the first studies a newly proposed constitutive equation was compared with the performance of empirical and physically based models. The modified Eyring equation was shown to be capable of accurately predicting the compressive yield stress over a strain rate range of 10^−4^–10^4^ s^−1^, and uniaxial tensile yield stress between 10^−3^–10^3^ s^−1^. Although this provided a fundamental basis for further understanding of their respective applications, during the thermoforming process the polymer is subjected to large, complex strain histories and is typically taken beyond their characteristic yield stress to ensure permanent forming of the product. It would therefore be desirable to understand and be able to model the material response beyond this point.

Recent work by Chen et al. [17] recognised the shortcomings of the aforementioned studies, with lack of consideration of the influence of temperature on modelling the post-yield flow stress of PEEK specimens. They initially attempted to model the compressive flow stress above strains of 0.05, given in previous literature [8], using a Johnson Cook (JC) model. It was shown that the JC model could accurately reproduce data at room temperature but at the highest temperature a 38% discrepancy was observable between measured and simulated data. This led to the authors creating a modified version of the JC model, by substituting an altered temperature term and re-calibrating. A maximum deviation of 12% from the experimental flow stress was simulated, with the resultant yield stress predictions correlating well over the strain rate range of 10^−4^–10^2^ s^−1^ and temperatures between 33 and 200 °C. Following on from this study, the authors furthered their research into the modelling of uniaxial extension of PEEK around the glass transition [12]. After low strain rate experimental testing (0.01–1 s^−1^) at temperatures between 100 and 150 °C the above-mentioned JC and modified JC models were calibrated as well as a second modified JC model devised to more accurately capture the coupling effect of strain rate and temperature [18], not considered in the previous models. The authors detailed that the original JC model was the most suitable in simulating uniaxial tensile stress-strain behaviour below the glass transition, with the modified models giving maximum deviations of 16% and 21%. Difficulties in modelling flow stress above T_g_ were documented and hypothesised to be as a result of changing structure within the polymer. It is of particular interest to note that the material model best suited for replicating uniaxial tensile behaviour is unsuitable for modelling compression of PEEK samples. This conclusion further strengthens the argument that in order for an accurate representation of material behaviour, the chosen material model must be calibrated to data at expected modes of deformation experienced during the process under investigation.

In order for accurate simulation of material behaviour during deformation an appropriate material model capable of capturing the observed material behaviour during stretching is vital. The physically based, three-dimensional Buckley material model was developed originally to the replicate nonlinear, temperature and strain rate dependent stress/strain behaviour of amorphous polyethylene terephthalate (aPET) near its T_g_ [19,20]. Several authors have detailed the suitability of the Buckley material model to further model thermoplastics above their respective T_g_ for many polymer forming processes [7,20,21,22]. Nixon et al. [23] adopted the Buckley material model for investigation into the forming behaviour of aPET subject to changing external forming parameters. The material model, previously calibrated through displacement-controlled biaxial testing [7], was implemented into a three dimensional finite element simulation of the stretch blow moulding process with the final part shape examined, amongst others. The authors [23] found strong correlation between the experimentally observed final part shapes and those predicted in simulations. By observing the biaxial stress–strain data recorded in a previous study [13] parallels can be drawn between the biaxial deformation behaviour observed for aPET and PEEK, thus warranting the evaluation of this material model to capture the nonlinear, viscoelastic behaviour of PEEK.

To the best of the author’s knowledge no previous studies exist in modelling the constitutive biaxial tensile deformation behaviour of PEEK subject to finite deformation. The importance of characterisation through this experimental technique is of particular concern in order to quantify and understand the material behaviour during thermoforming processes. The ultimate aim of this work was to accurately replicate the biaxial stress-strain behaviour of PEEK specimens under a variety of loading conditions through the following research objectives: (a) firstly a suitable material model is identified capable of reproducing the nonlinear viscoelastic constitutive response, (b) the chosen material model is modified to accommodate the post-yield anisotropic behaviour and necking behaviour, (c) then physically calibrated based on previously generated biaxial stress–strain data within the forming window, and (d) the final simulated material behaviour compared to the experimentally measured stress–strain behaviour above and below the glass transition.

## 2. Constitutive Modelling

### 2.1. Material Testing

**Figure 1:**
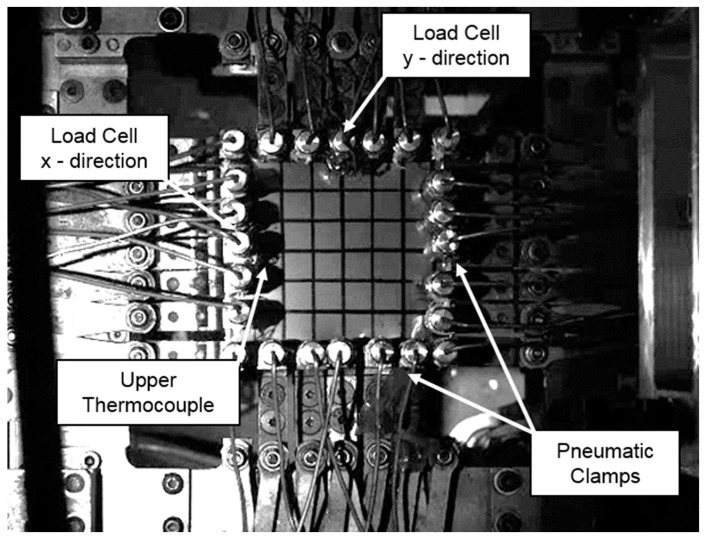
Annotated photo of poly-aryl-ether-ketones, poly(ether-ether-ketone) (PEEK) film mounted in Queen’s Biaxial Stretcher (QBS) [13].

**Figure 2:**
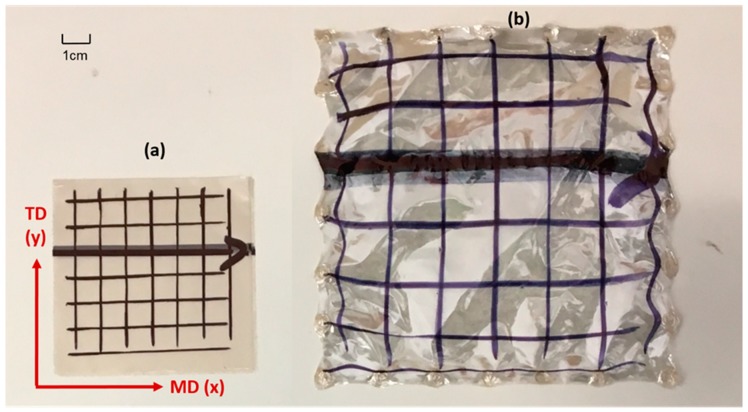
Equal Biaxial deformation of PEEK specimens (**a**) unstretched and (**b**) biaxially stretched to a ratio of 2 by 2 [13].

Extensive biaxial testing of PEEK samples has been conducted and presented in previous work [13], and hence a brief summary is presented here. Following Dynamic Mechanical Analysis of the PEEK films, of number average molecular weight equal to 45,000 g/mol, a formable temperature window spanning above and below the T_g_ of 150 °C was revealed (130–155 °C). Upon realization of this forming window, the 12 μm thick extruded PEEK films were biaxially stretched in the machine (MD) and transverse directions (TD) simultaneously at equal strain rates and displacements, detailed in previous work [13] at the conditions shown in Table 1. Samples were stretched using the purpose-built Queen’s Biaxial Stretcher (QBS)—specializing in the replication of temperature, deformation speeds and modes of deformation equivalent to those experienced during thermoforming and stretch blow moulding processes. The square specimens were held by 24 pneumatic clamps around its edges, shown in Figure 1, and heated to the specified temperature by two convection heaters: one placed above the sample and one below. After reaching the desired temperature the sample was simultaneously stretched in two directions by two servo-controlled motors, whilst a load cell located centrally in each axis recorded the force. The force and displacement data were then converted to true stress and nominal strain as described by O’Connor [24]. A typically sample stretched equal-biaxially is shown beside an unstretched counterpart in Figure 2. Planar biaxial characterization highlighted the non-linear viscoelastic stress–strain behaviour of the films, along with post-yield anisotropy at conditions equivalent to the forming process. The true stress–nominal strain data presented in the companion paper [13] concerning the in-plane biaxial deformation behaviour of PEEK provides the foundation in which the material model can be calibrated in a step-by-step approach, similar to that for aPET as presented by Buckley et al. [19,20].

### 2.2. Buckley Material Model

It is assumed in the Buckley material model that the total stress is from a contribution of two independent sources: a bond-stretching and conformational part—represented in 1D by a parallel network of a spring and dashpot in series. The former is described by a linear elastic spring, to model initial linear elasticity, and viscous Eyring formulation for an activated rate process representing the temperature and strain rate dependence of the yield stress. The latter is characterized by an Edwards–Vilgis spring and nonlinear dashpot to represent strain hardening and entanglement slippage between polymeric chains associated with being above the glass transition. The principal values of Cauchy stress (*σ_i_*) are represented through the addition of the individual contribution of the two sources as shown in Equations (1)–(3):

(1) $$\sigma_{i}=\sigma_{i}^{b}+\sigma_{i}^{c}\left(i=1,\;2,\;3\right),$$

(2) $$\sigma_{i}=s_{i}^{b}+K^{b}\sum_{i=1}^{3}\in_{i}+\sigma_{m0}^{b}+\sigma_{i}^{c},$$

(3) $$\sigma_{m}^{b}=K^{b}\ln\left(J\right)+\sigma_{m0}^{b},$$

where $\sigma_{i}^{b}$ and $\sigma_{i}^{c}$ are the bond-stretching and conformational stresses in the principal directions respectively. $s_{i}^{b}$ is the principal deviatoric bond stretching stress, $K^{b}$ is the bulk modulus, $\in_{i}$ is the principal true strain in each direction, $\sigma_{m0}^{b}$ is the stress at zero strain, $\sigma_{m}^{b}$ is the strain induced hydrostatic stress and J is the determinant of the deformation gradient tensor. Deviatoric stresses ($s^{b}$) within the bond stretching arm exhibit viscoelasticity in terms of the deviatoric true strains (e) by Equation (4):

(4) $$2G^{b}\frac{de}{dt}=\frac{ds^{b}}{dt}+\frac{s^{b}}{\tau },$$

where $G^{b}$ is shear modulus arising from bond stretching and τ is the relaxation time—obtained by shifting the linear viscoelastic relaxation time at a reference temperature and structure ($\tau_{0}^{*}$). The shift factors responsible are due to stress ($\alpha_{\sigma }$), structure ($\alpha_{s}$) and temperature ($\alpha_{T}$) using an Eyring formulation [25], Vogel–Tammann–Fulcher function [26,27,28] and Arrhenius equation [29], as shown in Equations (5)–(8):

(5) $$\tau =\tau_{0}^{*}\alpha_{0}\alpha_{s}\alpha_{T},$$

(6) $$\alpha_{\sigma }=\frac{V_{s}\tau_{oct}^{b}}{2RT}\frac{\exp\left(-\frac{V_{p}\Delta \sigma_{m}^{b}}{RT}\right)}{\sinh\left(\frac{V_{s}\tau_{oct}^{b}}{2RT}\right)},$$

(7) $$\alpha_{s}=\exp\left(\frac{C^{v}}{T_{f}-T_{\infty }}-\frac{C^{v}}{T_{f}^{*}-T_{\infty }}\right),$$

(8) $$\alpha_{T}=\exp\left[\frac{\Delta H_{0}}{R}\left(\frac{1}{T}-\frac{1}{T^{*}}\right)\right],$$

where $V_{s}$ is the shear activation volume, $V_{p}$ is the pressure activation volume, $\tau_{oct}^{b}$ is the isotropic invariant of the stress state, $\Delta \sigma_{m}^{b}$ is the mean stress, R is the universal gas constant, $C^{v}$ is the Cohen–Turnbull constant, $T_{\infty }$ is the Vogel temperature and $\Delta H_{0}$ is the activation enthalpy. T and $T_{f}$ are actual and fictive temperature, whilst the reference configurations of these variables are denoted by $T^{*}$ and $T_{f}^{*}$ respectively. In previous work concerning the deformation behaviour of polymers above their characteristic glass transition $T_{f}$ is assumed to equal *T* [20], but this cannot be assumed for materials deformed below T_g_. Post-yield softening is typically observed for this condition when stress is plotted against strain and is captured through variation of fictive temperature with visco-plastic strain, as detailed in Equation (9) [30]:

(9) $$T_{f}=T_{f0}+\Delta T_{f}\left[1-\exp\left(-\;{\left(\frac{{\bar{\varepsilon }}_{j}^{v}}{\varepsilon_{0}^{v}}\right)}^{r}\right)\right],$$

where $T_{f0}$ is the initial fictive temperature, $\Delta T_{f}$ is the change in fictive temperature due to rejuvenation, $\varepsilon_{0}^{v}$ is the strain range in which rejuvenation occurs, ${\bar{\varepsilon }}_{j}^{v}$ is the von Mises equivalent viscoplastic strain and *r* is a fitting parameter of order unity.

In the original material model the conformational stress, $\sigma_{i}^{c}$, is characterised by the hyperelastic Edwards–Vilgis model [31], representative of polymeric chains of finite length physically entangled with one another. The free energy function, $A^{c}$, of the Edwards–Vilgis model is defined by Equation (10):

(10) $$\begin{array}{l} A^{c}=\frac{N_{e}K_{B}T}{2}[\frac{\left(1+\eta \right)\left(1-\alpha^{2}\right)}{1-\alpha^{2}\sum_{i=1}^{3}\lambda_{i}^{n^{2}}}\overset{3}{\underset{i=1}{\sum }}\frac{\lambda_{i}^{n^{2}}}{1+\eta \lambda_{i}^{n^{2}}}\\ \;+\overset{3}{\underset{i=1}{\sum }}\ln\left(1+\eta \lambda_{i}^{n^{2}}\right)+\ln\left(1-\alpha^{2}\overset{3}{\underset{i=1}{\sum }}\lambda_{i}^{n^{2}}\right)], \end{array}$$

where *N_e_* is the density of entanglements, *K_B_* is Boltzmann’s constant (1.38 × 10^−23^ m^2^kg s^−2^ K^−1^), *η* is the parameter identifying the looseness of entanglements, *λ_i_^n^* is the network stretches in principal directions and α is the inextensibility of the entanglement network. The constitutive material model was further developed by Adams et al. [31] to incorporate entanglement slippage when above T_g_. The viscosity of this dashpot, γ, dictating the onset of ‘lock-up’ of molecular chains, is governed by Equation (11):

(11) $$\gamma =\gamma_{0}/\left(1-\frac{\lambda_{max}^{n}}{\lambda_{crit}^{n}}\right),$$

where $\lambda_{max}^{n}$ and $\lambda_{crit}^{n}$ are maximum principal and critical values of the network stretch, with $\gamma_{0}$ the initial viscosity of the dashpot. For further insight, an extensive description of the material model has already been detailed elsewhere in previous literature [19,20,32].

### 2.3. Existing Capabilities of Buckley Material Model

**Figure 3:**
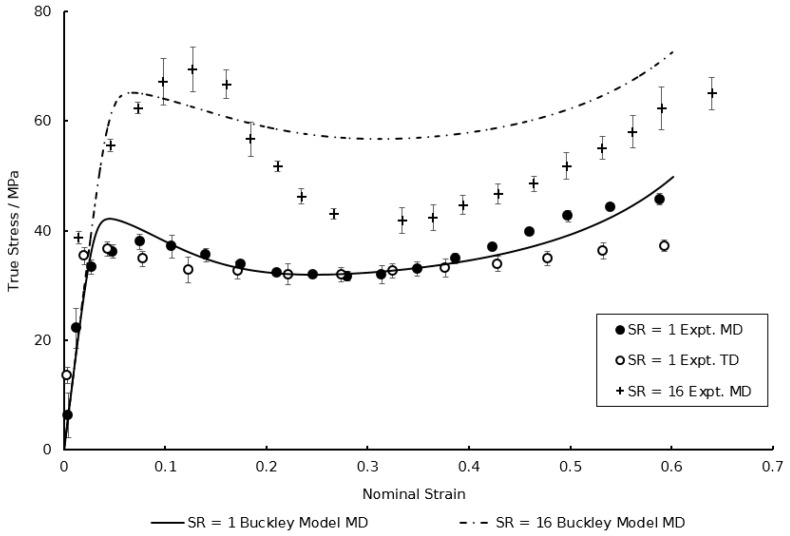
Comparison between simulated and experimental Equal Biaxial (EB) deformation behaviour of PEEK in machine and transverse directions at strain rates of 1 and 16 s^−1^ at a temperature of 130 °C.

Following calibration to the model described in Section 2.1, the specifics of which are detailed later, the simulated equal biaxial stress–strain behaviour in both material directions at a temperature of 130 °C and a strain rate of 1 s^−1^ is shown in Figure 3. Two major shortcomings are noticeable. Firstly, it is evident that stress levels post-yield in the machine direction (MD) are significantly greater than those observed in the transverse direction (TD)—equal to a 20% difference in total stress at failure between the two principal directions. The current capability of the traditional Buckley material model is unable to reproduce this phenomenon, assuming isotropic deformation after yielding. Second, is the inability to accurately capture the increased dip in stress after yielding, known as necking, shown in Figure 3 with biaxial deformation at the highest strain rate (16 s^−1^). In the current model no concern is given to the potential evolution of yield stress behaviour with strain rate. It is therefore necessary that modifications be made in order for accurate representation of the equal biaxial deformation of PEEK specimens.

### 2.4. Modification to the Material Model

As mentioned, one such limitation of the traditional Buckley material model is that associated in the calculation of isotropic conformational stresses by the current Edwards–Vilgis model. To accommodate the observed post-yield anisotropic behaviour during biaxial stretching of PEEK specimens, the anisotropic Holzapfel–Gasser–Ogden (HGO) material model, initially developed to imitate the anisotropic mechanical response of arterial walls during inflation tests [33], was chosen.

The isochoric strain energy function of the HGO model is calculated through superposition of an isotropic part, $\psi_{iso}$, associated with the non-collagenous matrix, and an anisotropic part due to the orientated fibres, $\psi_{aniso}$. The total strain energy per unit volume, ψ, is detailed by Equation (12):

$$\psi =\psi_{iso}+\;\psi_{aniso}$$

(12) $$=\mu {\left(I_{1}-3\right)}_{iso}+\frac{k_{1}}{k_{2}}{\left(\exp\left\{k_{2}\left[\left(1-\rho \right){\left(I_{1}-3\right)}^{2}+\rho {\left(I_{4}-1\right)}^{2}\right]\right\}-1\right)}_{aniso},$$

where $I_{1}$ is the first invariant of the right Cauchy–Green tensor, μ and $k_{1}$ are parameters given in MPa, and $k_{2}$ and ρ are dimensionless parameters. $I_{4}$ is a pseudo-invariant as defined by Equation (13):

(13) $$I_{4}=\lambda_{1}^{2}\cos^{2}\beta +\lambda_{2}^{2}\sin^{2}\beta \;\lambda_{1},$$

where β is the fibre angle measured between the MD axis and the direction of fibre alignment, with $\lambda_{1}$ and $\lambda_{2}$ equal to the total stretches in principal directions. The stress components in each direction are then calculated by Equation (14):

(14) $$\sigma_{11}=\lambda_{1}\frac{\delta \psi }{\delta \lambda_{1}},\;\;\;\sigma_{22}=\lambda_{2}\frac{\delta \psi }{\delta \lambda_{2}}.$$

Although clearly no fibres, collagenous or otherwise, are present within the biaxially stretched PEEK samples, one can confidently hypothesise that the observable differences in stress values seen post-yield between the MD and TD are due to the molecular alignment of polymeric chains in the underlying microstructure of the material. As a result of the analogy between the polymeric chains and the fibres, the fibre angle can be defined as that between the MD and direction of proposed chain alignment.

**Figure 4:**
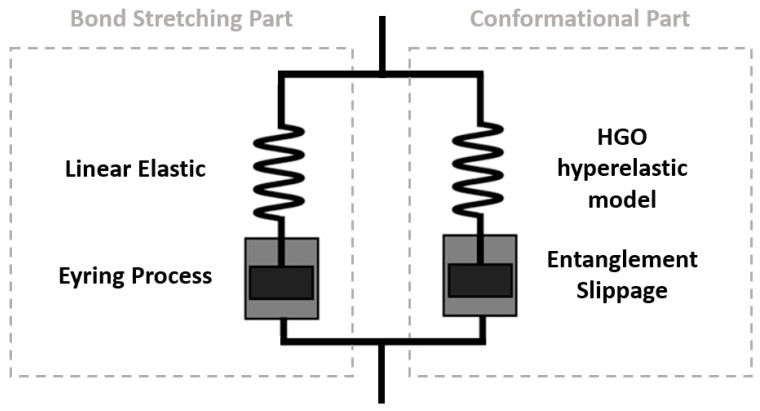
Spring and dashpot assembly of the proposed Buckley material model including modifications to the conformational part.

A representation of the modified Buckley material model can be seen in Figure 4. It is with this proposed model configuration that calibration will be performed using the extensive displacement-controlled biaxial data provided in previous work [13] over the wide temperature and strain rate window associated with the real-life thermoforming process.

## 3. Fitting of Constitutive Model

Calibration of the proposed material model was conducted using previously detailed biaxial data at strain rates between 1 and 16 s^−1^ and temperatures of 130–155 °C, for equal biaxial and constant width modes of deformation. From Equation (4) it can be seen that the shear modulus, G_b_, is necessary in modelling the initial stiffness response exhibited in the true stress–nominal strain curves. Assuming incompressibility and isotropy, correct for both material directions subject to small strains, the G_b_ of a given material can be commonly calculated as a third of the Young’s modulus. Between a nominal strain range of 0–0.02 the Young’s modulus of PEEK samples at 130 °C and a strain rate of 1 s^−1^ was found to be 750 MPa, corresponding to a value of 250 MPa for the shear modulus. It is noted that the tensile elastic modulus of PEEK specimens appears to be unaffected by temperature, deformation rate and material orientation [13].

### 3.1. The Eyring Process in the Bond Stretching Component

**Figure 5:**
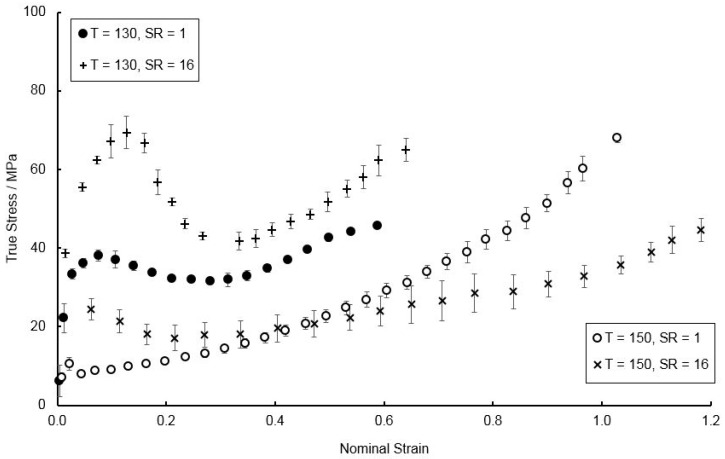
Influence of strain rate on resultant stress-strain behaviour at temperatures of 130 and 150 °C in the machine direction (MD).

The dependency of the yield stress of thin PEEK specimens as a function of temperature and strain rate, is evident from previous literature [13] and highlighted in Figure 5.

**Figure 6:**
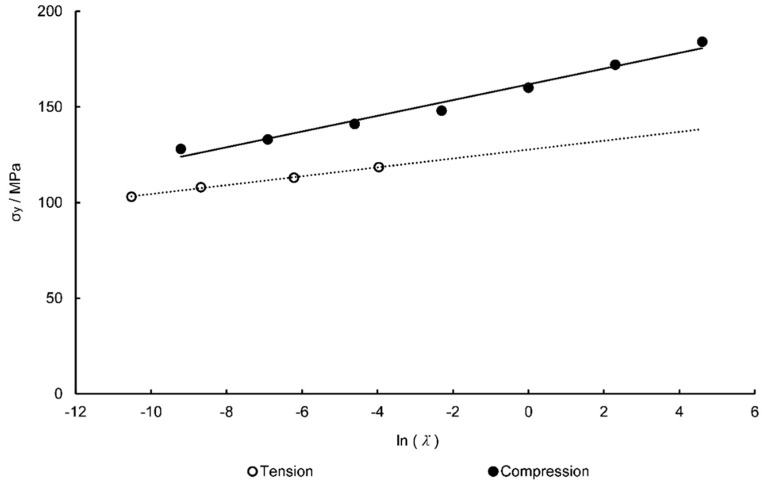
Tensile and compressive yield stresses plotted against natural log of true strain rate [11].

One such way of modelling the deformation rate dependency of yield stresses is through the use of the Eyring activation rate process. Buckley et al. [20] previously concluded that the contribution of bond stretching on the total stress can be described by Equation (15):

(15) $$\frac{\sigma_{y}}{T}=\frac{6R}{2\left(1+\xi \right)V_{p}+\sqrt{2}\sqrt{1-\xi +\xi^{2}}V_{s}}\left[\ln\left(\frac{1}{\lambda_{y}}\frac{d\lambda }{dt}\right)+\ln\left(\frac{\sqrt{2}\sqrt{1-\xi +\xi^{2}}\mu_{0}V_{s}}{\left(2-\xi \right)RT}\right)\right],$$

where $V_{s}$ and $V_{p}$ are shear and pressure activation volumes, $\sigma_{y}$ is the true stress at yield, T is the absolute temperature, λ is the total stretch, R is the universal gas constant, $\mu_{0}$ is the limiting viscosity and ξ is a parameter concerned with the biaxial stretching conditions, governed by Equation (16):

$$\frac{1}{\lambda_{2}}\frac{d\lambda_{2}}{dt}=\frac{\theta }{\lambda_{1}}\frac{d\lambda_{1}}{dt}$$

(16) $$\xi =\frac{2\theta +1}{\theta +2},\;\xi \in \left[0,1\right],$$

where θ is a ratio of the in-plane natural strain rate. Values for $V_{s}$ and $V_{p}$ were computed using a similar methodology as Dooling et al. [21]. Using previous work by Rae et al. [8], the ratio of uniaxial tensile and compressive yield stresses of PEEK were plotted and shown in Figure 6. The relationship between the activation volumes and ratio of tensile and compressive yield stresses are given by Equation (17) [34]:

(17) $$\frac{\sigma_{yc}}{\sigma_{yt}}=\frac{1+\sqrt{2}V_{p}/V_{s}}{1-\sqrt{2}V_{p}/V_{s}}.$$

**Figure 7:**
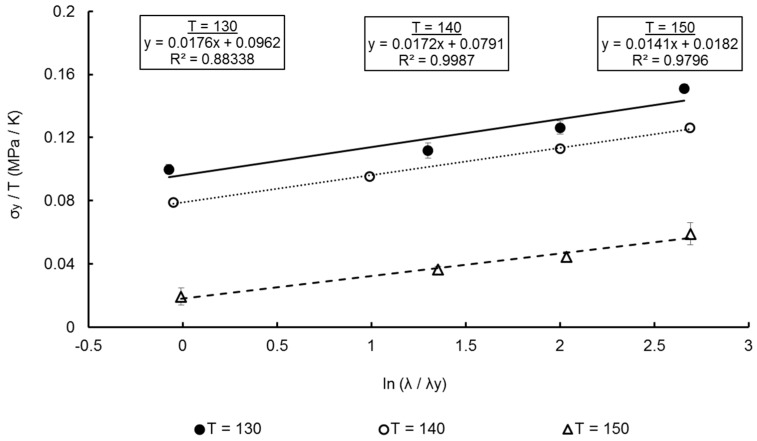
Eyring plots for EB deformation of PEEK between temperatures of 130–150 °C, at a nominal strain rate range of 1–16 s^−1^.

From Figure 3, at a chosen reference strain rate of 1 s^−1^, the ratio of yield stresses is 1.26—close to the value of 1.3 widely discerned for many glassy polymers [21]. From Equation (17) the ratio of activation volumes is calculated to be 0.0821. From Equation (15) when the equal-biaxial true yield stress over temperature, σyT, is plotted against the natural logarithm of the true strain rate at yield, $\ln\left(\frac{1}{\lambda_{y}}\frac{d\lambda }{dt}\right)$, a linear relationship is noted as observed in Figure 7. Taking an average of the gradients of the Eyring plots for each temperature, equal to 0.0167 MPa K^−1^, the activation volumes can be calculated from Equation (15), with values of 1.714 × 10^−3^ m^3^ mol^−1^ and 1.407 × 10^−4^ m^3^ mol^−1^ determined for $V_{s}$ and $V_{p}$ respectively.

**Figure 8:**
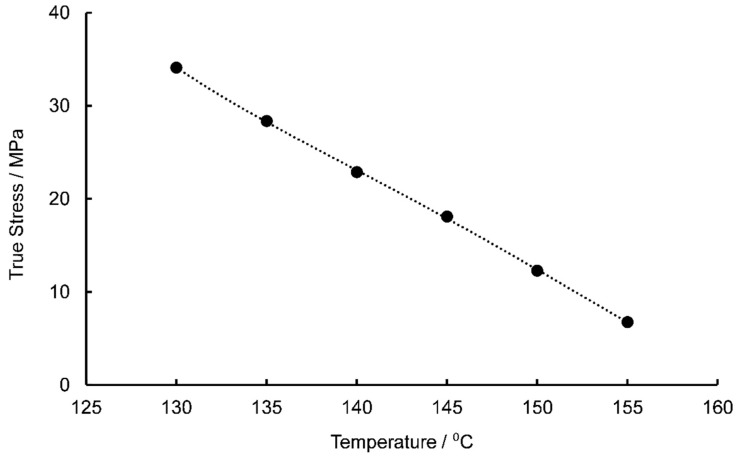
Isometric stress-temperature plot between a temperature range of 130–150 °C, at a strain rate of 1 s^−1^.

The temperature effect on decreasing yield stress is incorporated into the bond stretching component of the Buckley material model through the limiting viscosity, $\mu_{0}$, is given by Equation (18):

(18) $$\mu_{0}=\sqrt{2}\frac{\lambda }{\dot{\lambda }}\frac{RT}{V_{s}}\sinh\left(\frac{\sqrt{2}V_{s}\sigma^{b}}{6RT}\right)\exp\left(\frac{2V_{p}\sigma^{b}}{3RT}\right),$$

where $\sigma^{b}$ is the bond stretching stress. Traditional methods for determining this variable require the identification of a rubber-like plateau within isometric true stress-temperature plots. It is at the temperature of this plateau that the contribution to total stress by bond stretching is assumed to decrease to zero and the total stress is now solely a product of the conformational arm. In this work the true stresses were established at a nominal stretch of 1.2, just after yielding, for EB deformation at a nominal strain rate of 1 s^−1^ in order to minimise any potential self-heating effects. The isometric stress against temperature is shown in Figure 8.

As can be seen in Figure 8, the total true stress levels follow an almost linear decrease with increasing temperature with a lack of an apparent plateau region evident. In this case the bond stretching stress is assumed to be fully relaxed at the highest temperature of 155 °C, therefore the rubber-like stress, $\sigma^{c}$, assumed to be independent of temperature was considered to be equal to the total stress at a temperature of 155 °C (6.77 MPa). The bond stretching stresses can then be calculated by subtracting the rubber-like stress from the total stress (Equation (1)). Values of $\sigma^{b}$ at each temperature were substituted into Equation (18) to calculate the limiting viscosity for each temperature in the forming window, shown in Table 2.

Modelling this viscosity on specimen temperature is accounted for by the previously mentioned Vogel–Fulcher–Tammann and Arrhenius functions, as shown in Equation (19):

(19) $$\mu_{0}=\mu_{0}^{*}\exp\left(\frac{C_{v}}{T_{f}-T_{\infty }}-\frac{C_{v}}{T_{f}^{*}-T_{\infty }}+\frac{{\Delta H}_{0}}{RT}-\frac{{\Delta H}_{0}}{RT^{*}}\right).$$

**Figure 9:**
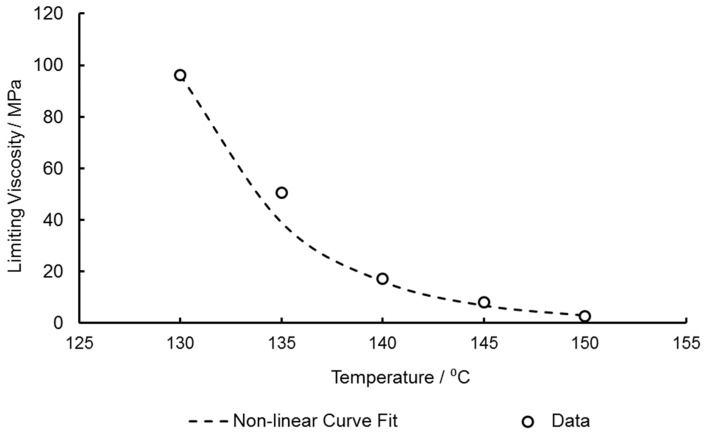
Plot of limiting viscosity against temperature showing non-linear curve fitting to Vogel– Fulcher–Tammann and Arrhenius functions.

A least squares curve fitting procedure was performed to characterise these parameters, with $\mu_{0}^{*}$ equal to 92.66 MPa—the limiting viscosity at the reference temperature of the characterisation window, $T^{*}$, of 130 °C (Table 1). For this fitting procedure, for temperatures below the glass transition, $T_{f}$ is preliminarily considered to be equal to the previously identified T_g_ of PEEK (150 °C), until temperatures above this in which the fictive temperature is assumed to be equal to the specimen temperature. Curve fitting resulted in values of ${\Delta H}_{0}$ = 301.2 kJ mol^−1^, $T_{\infty }$ = 465 K, and $C_{v}$ = 399 K, producing the plot of limiting viscosity against temperature as seen in Figure 9.

### 3.2. Evolution of Fictive Temperature in the Bond Stretching Part

**Figure 10:**
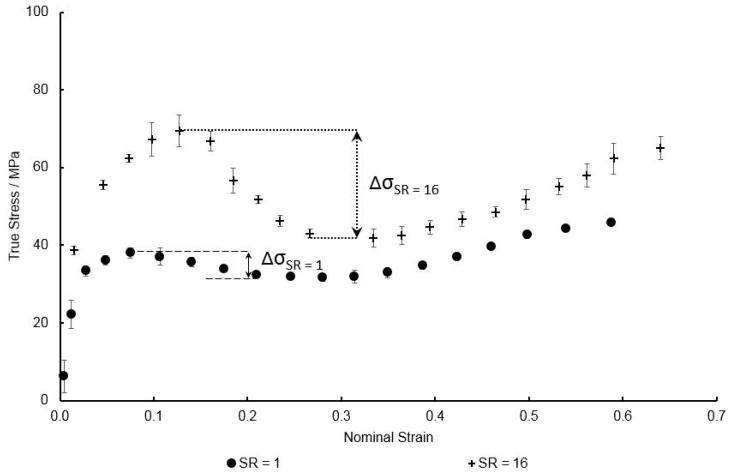
Observed variation drop in post-yield stress with increasing strain rate at a temperature of 130 °C.

**Figure 11:**
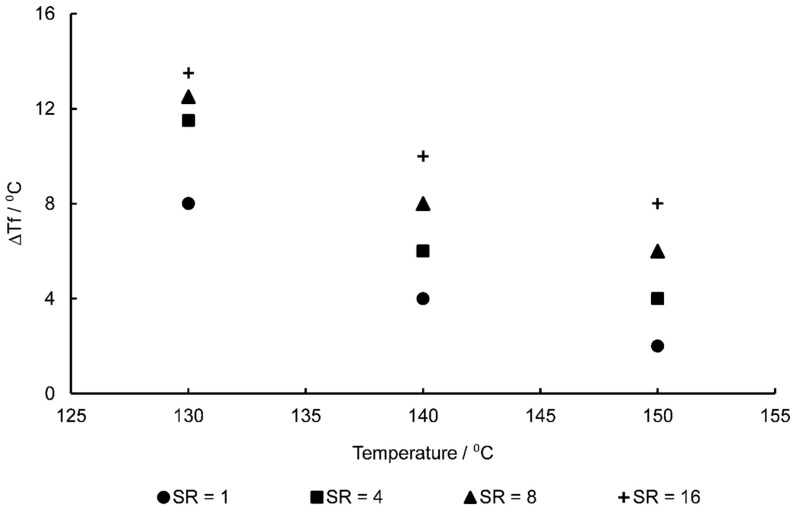
∆*Tf* against temperature for EB stretching of PEEK.

As mentioned in the previous section the evolution of fictive temperature, $T_{f}$, is important in capturing post-yield stress-softening effects when near the glass transition—the evolution of this variable being primarily dependent on the four parameters as outlined in Equation (9). Initial fitting of these to reference EB data at a temperature of 130 °C and a deformation rate of 1 s^−1^, yielded values of $T_{f0}$ = 419 K, $\Delta T_{f}$ = 7 K, $\varepsilon_{0}^{v}$ = 0.2, and r = 1.6. As observed in previous work the biaxial stress–strain curves of PEEK [13], with increasing strain rate creating a larger and broader decrease in post-yield stress levels (Figure 10) whilst increasing temperature is correlated with a decrease in these stresses. Previous work in modelling the deformation behaviour of glassy polymers has noticed the complexity of capturing the evolution of $T_{f}$ with increasing strain rate [30]. By probing Equation (9) the parameter $\Delta T_{f}$ was identified as that responsible for the magnitude of the drop observed in post-yield stresses with increasing values of $\Delta T_{f}$ producing elevated values of $T_{f}$—resulting in a softer material response after yielding and thus, lower associated true stress levels. Individual values of $\Delta T_{f}$ were then manually fitted to each test case of varying combinations of temperature and strain rate to best represent the experimental data, with Figure 10 showing this dependence of $\Delta T_{f}$ on strain rate and temperature. It is clear from the Figure that with increased strain rate the value of $\Delta T_{f}$ is seen to increase, correlated with an increased dip in stresses associated after yielding occurs, with the opposite being true with increasing specimen temperature–as shown when individually plotted in Figure 11.

**Figure 12:**
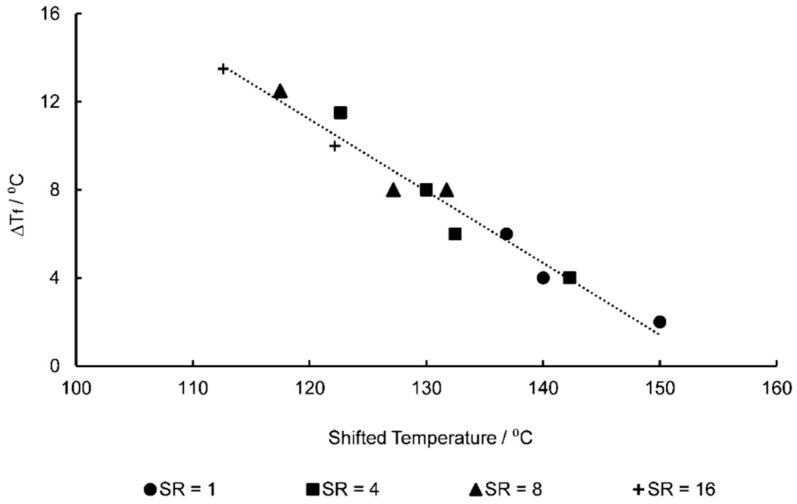
∆*T_f_* against shifted temperature following use of time-temperature superposition.

One such method of potential implementation of this $\Delta T_{f}$ evolution with strain rate and temperature is through the method of time-temperature superposition (TTS). Equation (20) demonstrates the calculation of a shift factor, α, with associated strain rate, $\dot{\varepsilon }$:

(20) $${log}_{10}\left(\alpha \right)=\frac{C_{1}\left(\dot{\varepsilon }-{\dot{\varepsilon }}_{ref}\right)}{C_{2}+\dot{\varepsilon }-{\dot{\varepsilon }}_{ref}},$$

where $C_{1}$ and $C_{2}$ are constants, and ${\dot{\varepsilon }}_{ref}$ is the reference strain rate chosen as 1 s^−1^. The constants in Equation (20) were fitted in order to shift the temperature data above a strain rate of 1 s^−1^ in Figure 10 intending to create a linear relationship of $\Delta T_{f}$ with shifted temperature. Values of −0.08915 and 5.905 for $C_{1}$ and $C_{2}$ respectively were derived resulting in Figure 12—a master curve of $\Delta T_{f}$ against shifted temperature, taking into account the rate of deformation. A strong linear relationship is exhibited in which an R^2^ value of 0.9637 is exhibited. This relationship is defined by Equation (21), with shifted temperature in Kelvin:

(21) $$\Delta T_{f}=-0.2525\;.\;T_{shifted}+109.$$

### 3.3. Calibration of HGO Model in the Conformational Part

**Figure 13:**
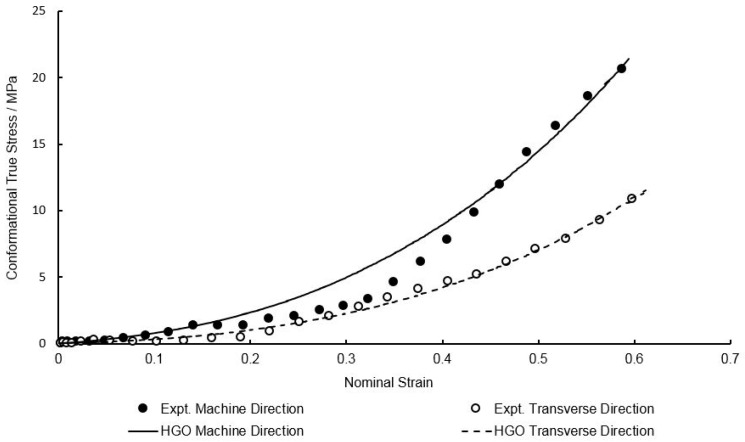
Non-linear curve fitting of Holzapfel–Gasser–Ogden (HGO) model to conformational stresses at a temperature of 130 °C and strain rate of 1 s^−1^

As previously highlighted in Equation (1) the total stress is a result of the addition of two components: the bond stretching stress and conformational stress. In the traditional Buckley material model the conformational stress is predicted by an isotropic Edwards–Vilgis formulation, but as already discussed these current methods are unsuitable for the biaxial deformation behaviour observed with the extruded PEEK sheets used in our studies—which clearly demonstrate some orientation in the MD, demonstrated through the larger stress response in the MD direction compared to the TD. In order to model the post-yield deformation behaviour the simulated bond stretching stress must be subtracted from the total experimental stress, using the characterised parameters derived in the previous section. These equal-biaxial rubber-like stresses are shown in Figure 12 in the machine and transverse directions, at the reference temperature of 130 °C and strain rate of and 1 s^−1^. A least squares curve fitting procedure was then conducted to best fit the parameters in Equation (12) to the experimental conformational stresses, giving values of μ = 1.00 MPa, $k_{1}$ = 2.16 MPa, $k_{2}$ = 1.50 × 10^−2^, ρ = 0.585 and β = 4.02 × 10^−11^ ⁰. The generated conformational stresses by the characterised HGO model is shown in Figure 13 at a temperature of 130 °C and strain rate of 1 s^−1^.

### 3.4. Modelling Entanglement Slippage in the Conformational Part

In an extension to the constitutive model, Adams et al. [32] considered the inclusion of slippage to model the deformation behaviour of aPET above T_g_. The total conformational stretch is considered to be multiplicative combination of network, $\lambda^{n}$, and slippage stretches, $\lambda^{s}$, expressed by Equation (22):

(22) $$\lambda_{i}=\lambda_{i}^{n}\;\lambda_{i}^{s}\;\left(i=1,\;2,\;3\right).$$

**Figure 14:**
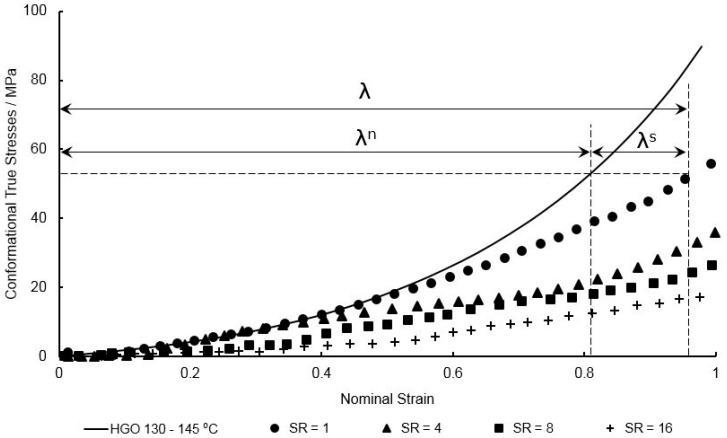
Comparison between conformational stresses in the MD of EB stretching of PEEK specimens for strain rates between 1 and 16 s^−1^ at a temperature of 150 °C, and simulated HGO network stresses, accurate for conformational stresses simulated at temperatures of 130–145 °C.

Biaxial stretching of PEEK at all strain rates and temperatures equal to and above the T_g_ creates a prolonged region of flowing behaviour creating a delay in strain hardening from occurring. It is assumed that the conformational stresses simulated by the HGO model experience no slip. From Equation (22) the slippage stretch can be calculated by division of the total stretch by the network stretch at equal levels of true stress, as highlighted in Figure 14 for a strain rate of 1 s^−1^ at a temperature of 150 °C.

**Figure 15:**
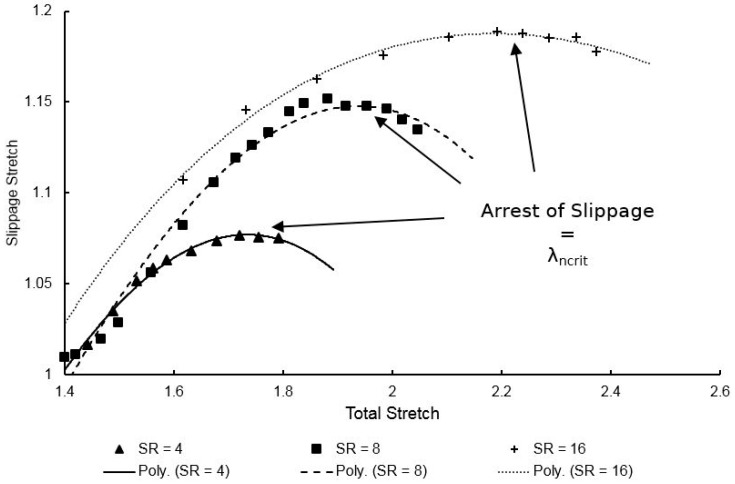
Slippage stretch plotted against total stretch for strain rates between 4 and 16 s^−1^, with fitted polynomials of order 3.

**Figure 16:**
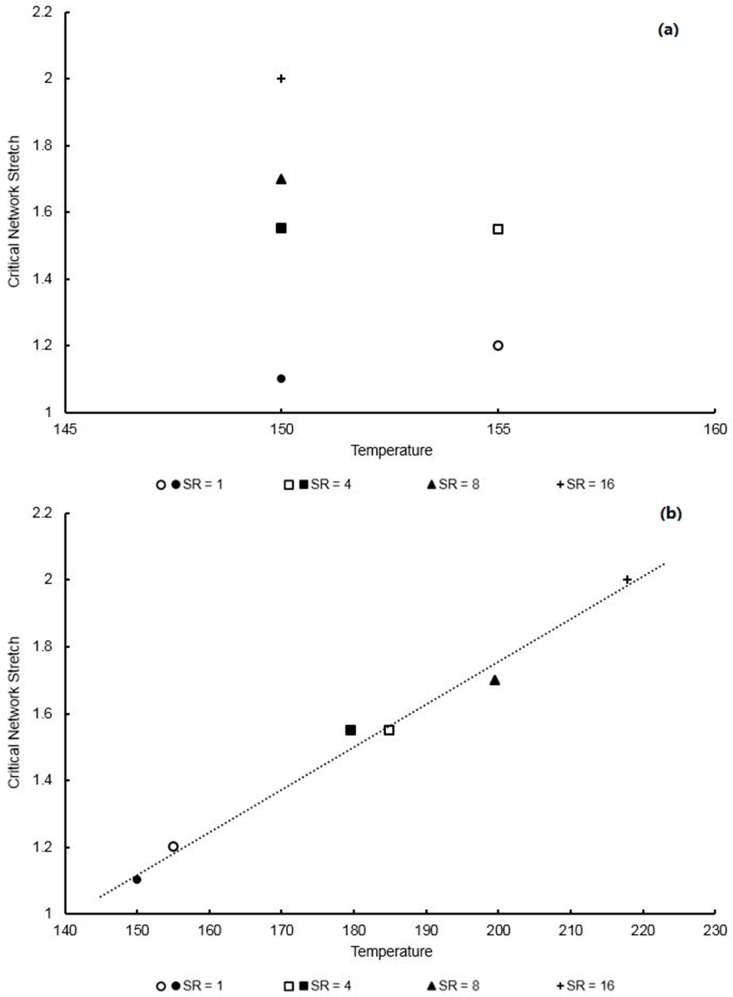
(**a**) Critical network stretch against temperature and (**b**) critical network stretch against shifted temperature following time-temperature superposition (TTS), between strain rates of 1–16 s^−1^ for EB stretching in the machine direction.

By plotting the slippage stretch against total stretch, a parameter known as the critical network stretch, $\lambda_{crit}^{n}$, can be found—the value at which entanglement slippage is considered to have fully arrested, ceasing flow behaviour and allowing the strain hardening process to occur. When a polynomial is fitted to the data, as shown in Figure 15, $\lambda_{crit}^{n}$ is defined as the point where the slippage stretch rate is equal to zero and derived when the gradient of the polynomial is horizontal. The critical network stretch is then found by division of the total stretch by the slippage stretch at this point, as defined by Equation (22). Plotting $\lambda_{crit}^{n}$ as a function of temperature and strain rate yields the plot observed in Figure 16, with increasing temperature and deformation rate inducing a proportional effect on the critical network stretch. Data at a temperature of 155 °C regarding strain rates above 4 s^−1^ proved difficult due to dynamic oscillatory effects appearing on the stress–strain plots as a result of dramatically softened material behaviour and is therefore not included.

Adoption of the TTS method was chosen, similar to that used in Section 3.2 to create a linear relationship between shifted temperature and critical network stretch. Following a least squares curve fit of the data shown in Figure 16, values of 0.2132 and 6.469 were characterized for $C_{1}$ and $C_{2}$ respectively in Equation (20). A linear relationship of R^2^ value equal to 0.986 is given in Equation (23), relating the critical network stretch and shifted temperature with little variation seen between material directions:

The slippage viscosity as a function of temperature, $\gamma_{0}$, as seen in Equation (23) is modelled by a Vogel–Tammann–Fulcher relationship [26,27,28]:

(23) $$\gamma_{0}=\gamma_{0}^{*}\exp\left(\frac{C^{s}}{T-T_{\infty }}-\frac{C^{s}}{T^{*}-T_{\infty }}\right).$$

**Figure 17:**
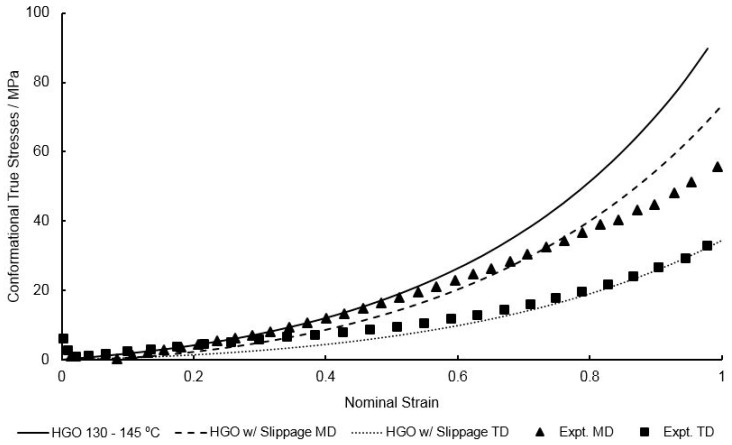
EB conformational stresses at a temperature of 150 °C and strain rate of 1 s^−1^ following fitting of slippage constants.

Similar to Equation (7), $C^{s}$ and $T_{\infty }$ are the Cohen–Turnbull constant and Vogel temperature governing the temperature dependency of the dashpot. $\gamma_{0}^{*}$ is the slippage viscosity at the reference temperature, $T^{*}$ and must also be direction dependant due to anisotropy observed when stretching samples. These parameters were fitted to post-T_g_ conformational stresses between deformation rates of 1–16 s^−1^, with the values of best fit equal to $C^{s}$ = 3296.50 K, $T_{\infty }$ = 5.95 K and $\gamma_{0}^{*}$ = 1.68 MPa and 0.20 MPa for the machine and transverse directions. Fits for the conformational stresses in both the MD and TD at a temperature of 150 °C are shown in Figure 17.

## 4. Modified Model Prediction and Discussion

The characterised material parameters of the Buckley material model, including modifications, are listed in Table 3.

**Figure 18:**
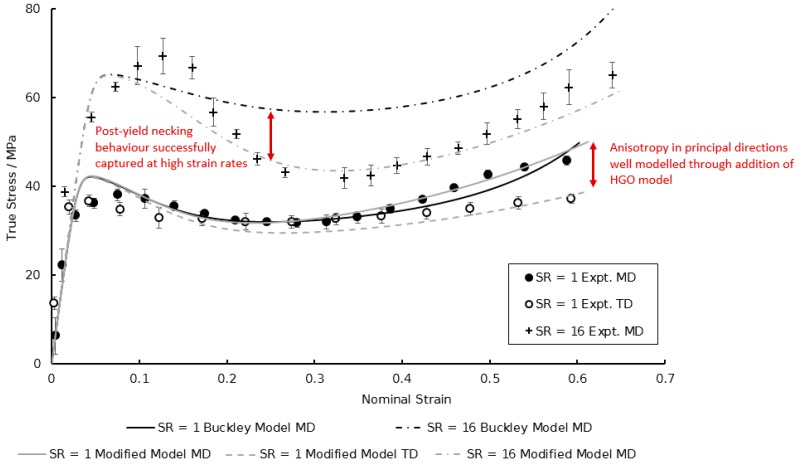
Comparison between simulated and experimental EB deformation behaviour of PEEK in machine and transverse directions at strain rates of 1 and 16 s^−1^ at a temperature of 130 °C.

The biaxial deformation behaviour can be predicted by the modified Buckley material model by employing the above characterised constants under differing combinations of experimental variables typical to those observed within the processing window. A comparison of the modified model with the original Buckley material model, the capabilities of which previously detailed in Figure 3, is now shown in Figure 18. The accuracies of the predicted material deformation behaviour compared with those observed experimentally are quantitatively analysed through calculation of the normalised root mean square error (NRMSE). This error, expressed as a percentage, is calculated by Equations (24) and (25):

(24) $$\%\;\mathrm{NRMSE}=\left[\frac{RMSE}{y_{max}-y_{min}}\right]\;\times \;100\%,$$

(25) $$\mathrm{RMSE}=\sqrt{\frac{\sum_{i=1}^{n}{\left(\hat{y_{t}}-y_{t}\right)}^{2}}{n}},$$

where $\hat{y_{t}}$ is the simulated stress value as predicted by the model and $y_{t}$ is the experimental stress value over the total number of strain data points, n. Table 4 details the %NRMSE values comparing the predictions of the modified model to the original Buckley model shown in Figure 18.

The improved ability of the proposed modified Buckley material model is in Table 4. At a strain rate of 1 s^−1^, the original and modified Buckley material model configurations are comparable in the machine direction, it is only when probing larger strain rates and material directions that the superiority of the modified model is demonstrated. In the transverse direction the associated errors with the original Buckley model is markedly greater, with the addition of the HGO hyperelastic element proving pivotal in replicating the post-yield anisotropic response of the PEEK films subject to biaxial loading. Previous use of the material model also showed no consideration of modelling the rate dependency of the post-yield dip in stress associated with necking below the T_g_, as shown in the machine direction at a strain rate of 16 s^−1^ with an error of almost 20%. When dealing with a combination of these two factors of high strain rates in the transverse direction, the error values seem to be compounded—with %NRMSE reaching values as large as 36%, warranting the adoption of the HGO conformational element and TTS for the accurate modelling of the specific material deformation behaviour.

**Figure 19:**
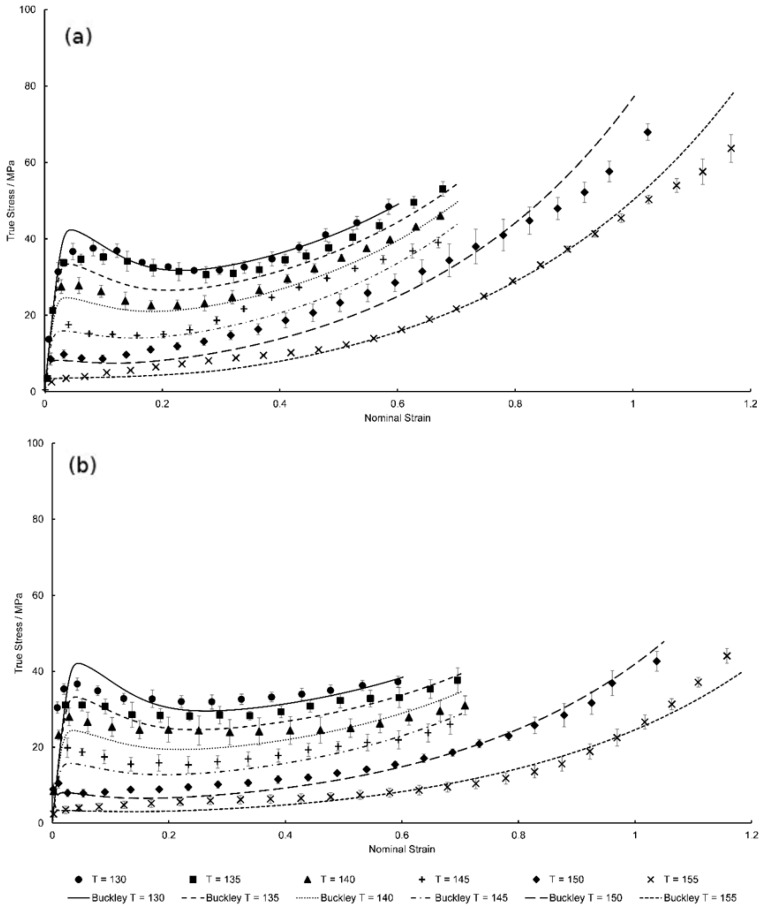
Predicted EB deformation behaviour by modified Buckley model for temperatures between 130 and 155 °C in the (**a**) machine direction and (**b**) transverse direction.

Figure 19 demonstrates the temperature dependence of equal-biaxial deformation in the machine and transverse directions respectively, at the reference strain rate of 1 s^−1^. The suitability of the modified Buckley model is clear from the Figure with initial stiffness, yielding, necking, and strain hardening stages of the biaxial deformation behaviour seen to be simulated well with only slight inaccuracies shown by underestimations of predicted yield stress levels at temperatures of 135 and 140 °C—16.3% and 10.1% deviation respectively. This correlates with the plot shown in Figure 9, where the modelled limiting viscosity governing the yield stress at these temperatures is less than the experimental values causing stress levels after yield to be slightly lower than those seen experimentally.

**Figure 20:**
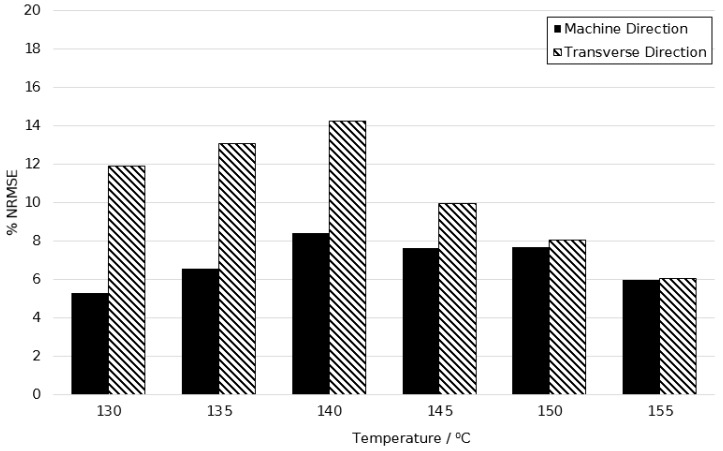
Normalised root mean squared error between simulated and experimentally observed stress–strain behaviour between temperatures of 130 and 155 °C, at a strain rate of 1 s^−1^.

Figure 20 shows the %NRSME values over the temperature range of 130–155 °C at a strain rate of 1 s^−1^. The average error between predicted and actual stress plots were 6.92% and 10.52% in principal directions respectively, showing simulations were more accurate in the machine direction. A minimum error of 5.29% at 130 °C and maximum of 14.20% at a temperature of 140 °C is in agreement with the above hypothesis concerning the modelling of limiting viscosity at this temperature.

Post-yield stress disparities are visually correlated appropriately with the observed biaxial deformation behaviour of PEEK specimens, highlighting the applicability and future promise of the HGO model in capturing anisotropic effects at all temperatures considered. Simulation of slippage at temperatures at the reference strain rate above the glass transition is captured effectively through use of TTS. Although minor deviation is only observed once above a strain of 1, overall the characterised material model is seen to qualitatively agree with the temperature dependency at all stages of the equal-biaxial deformation behaviour of PEEK.

**Figure 21:**
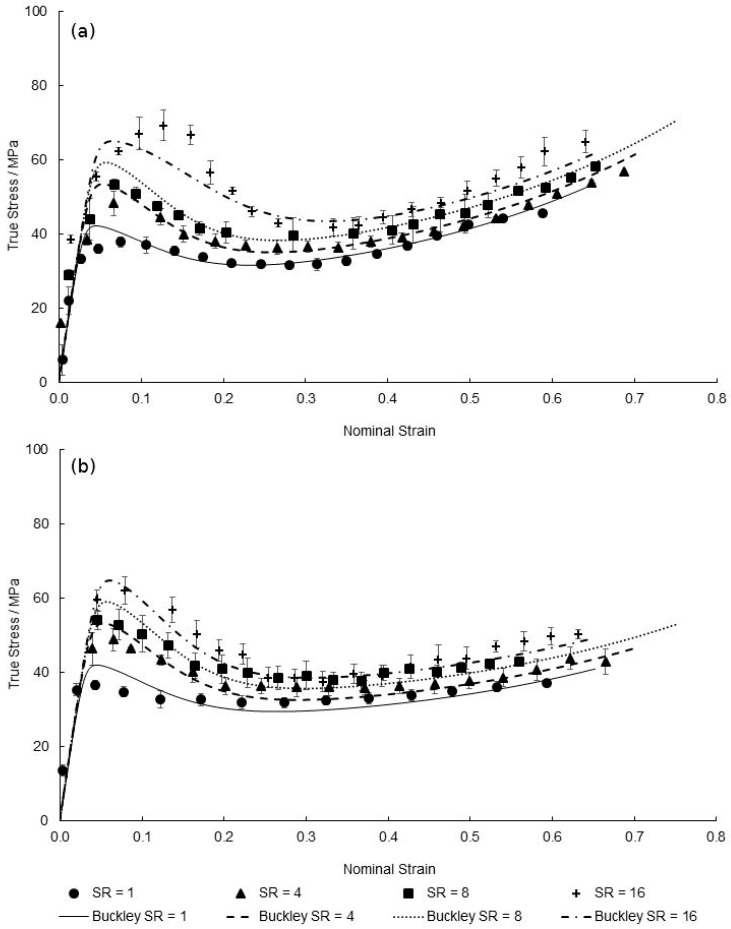
Predicted EB deformation behaviour by modified Buckley model for strain rates between 1 and 16 s^−1^, at a temperature of 130 °C in the (**a**) machine direction and (**b**) transverse direction.

**Figure 22:**
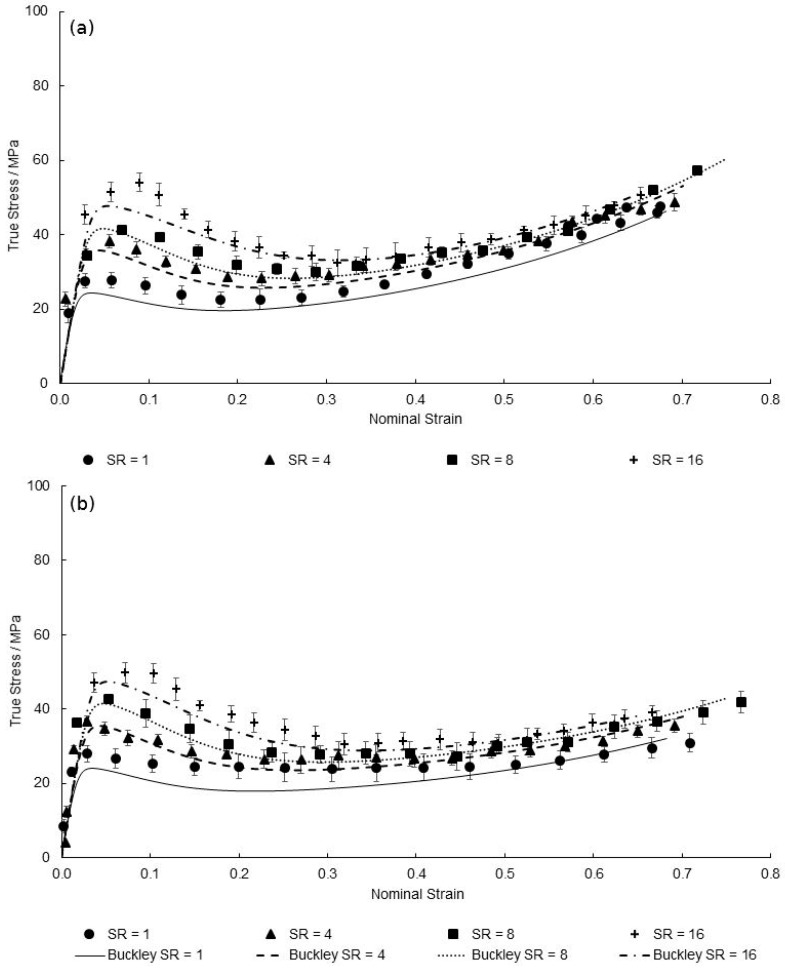
Predicted EB deformation behaviour by modified Buckley model for strain rates between 1 and 16 s^−1^, at a temperature of 140 °C in the (**a**) machine direction and (**b**) transverse direction.

**Figure 23:**
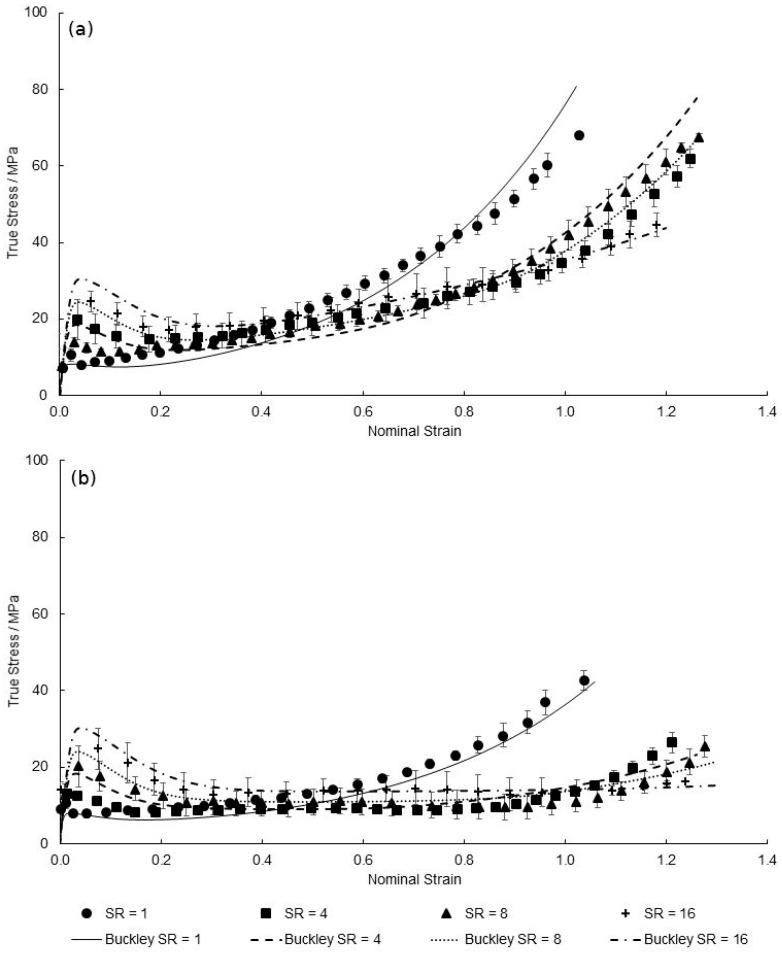
Predicted EB deformation behaviour by modified Buckley model for strain rates between 1 and 16 s^−1^, at a temperature of 150 °C in the (**a**) machine direction and (**b**) transverse direction.

Figure 21, Figure 22 and Figure 23 show the predicted true stress–nominal strain behaviour of PEEK specimens between strain rates of 1–16 s^−1^, at temperatures of 130–150 °C in both directions. Again, a good qualitative fit is exhibited by the characterised material model in replicating the aforementioned four stages of the deformation behaviour at elevated strain rates. Any differences observed between simulated and measured behaviour is typically focussed on modelling yield stress levels at the highest strain rate of 16 s^−1^, with maximum percentage differences of 6.28%, 12.2% and 12.7% recorded at the yield points of 130, 140, and 150 °C, respectively. By reviewing the Eyring plots in Figure 7 it is important to note that the values for $V_{s}$ and $V_{p}$, responsible for the magnitude in yield stresses, were calculated from an average of the individual gradients of the temperature plots. As the mean value of 0.0167 MPa K^−1^ was chosen, which is less than the value of the gradients at 130 and 140 °C but greater than that at 150 °C, this explains the simulated underestimation in yield stresses at a strain rate of 16 s^−1^ shown at temperatures of 130 and 140 °C, yet an overestimation for 150 °C at the highest strain rate. Although a larger value for the gradient to calculate the activation volumes would accommodate a more representative prediction for temperatures 130 and 140 °C, an average of all temperatures is used in order to ensure accurate modelling over the entire processing window.

**Figure 24:**
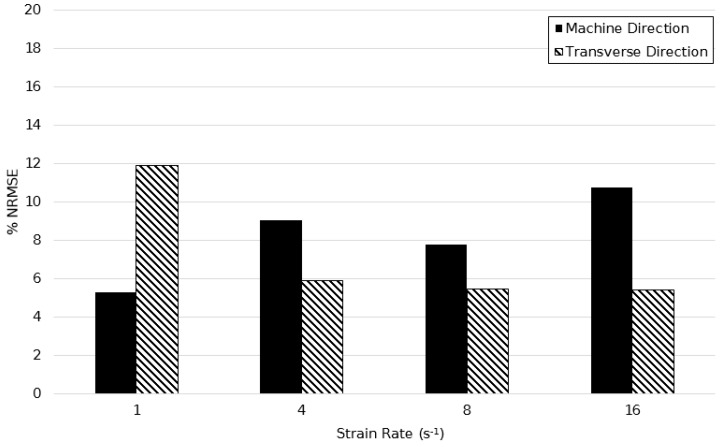
Normalised root mean squared error between simulated and experimentally observed stress–strain behaviour between strain rates of 1–16 s^−1^ at a temperature of 130 °C.

**Figure 25:**
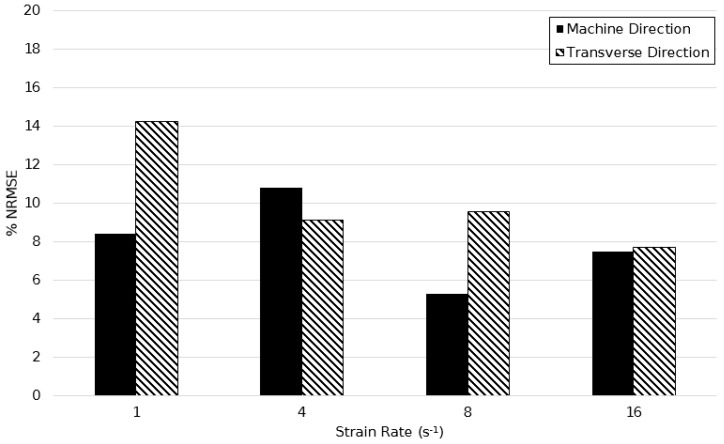
Normalised root mean squared error between simulated and experimentally observed stress–strain behaviour between strain rates of 1–16 s^−1^ at a temperature of 140 °C.

**Figure 26:**
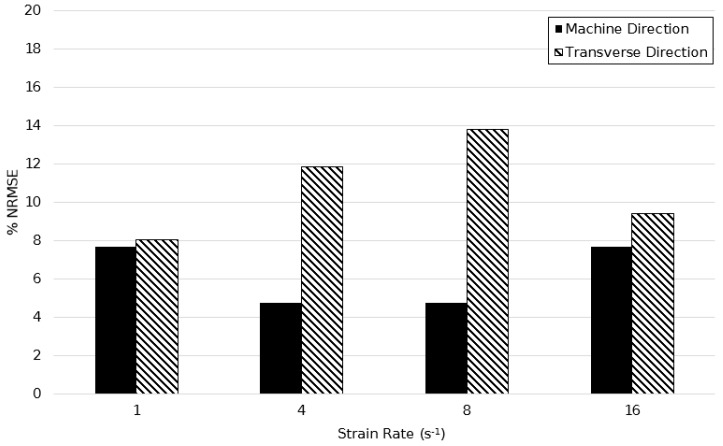
Normalised root mean squared error between simulated and experimentally observed stress–strain behaviour between strain rates of 1–16 s^−1^ at a temperature of 150 °C.

Figure 24, Figure 25 and Figure 26 show the calculated %NRSME values over the strain rate range of 1–16 s^−1^ at temperature intervals of 130, 140, and 150 °C in machine and transverse directions. The figures demonstrate the quantitative agreement of the modified model over the broad processing range, with maximum error levels not seen to exceed 15%. In general, the deformation behaviour was modelled more accurately in the machine direction—the maximum average percentage difference in principal directions being equal to only 4%.

## 5. Conclusions

In this work the constitutive biaxial deformation behaviour of thin PEEK specimens was successfully modelled extending extensive displacement-controlled biaxial tests over a temperature window of 130–155 °C and strain rate range of 1–16 s^−1^. The capability of the original Buckley material model was shown to capture linear elastic, and yielding behaviour associated with that observed during in-plane biaxial tests, although could not account for anisotropic effects revealed after yielding between the principal directions. The Holzapfel–Gasser–Ogden hyperelastic model was seen to successfully replicate the anisotropic stress-strain response of arterial tissues, and as a result, was substituted in place of the Edwards–Vilgis hyperelastic element within the conformational arm. The post yield divergence in true stresses observed in equal-biaxial tests of PEEK specimens was successfully modelled, resulting in an almost 10% decrease in the error levels associated with the inability to replicate this post-yield anisotropy in the original model, at a temperature of 130 °C. When concerning the discernible dependencies of the magnitude in post-yield stress declines at large strain rates, as suggested in previous literature, the time-temperature superposition principal has shown promise in effectively computing the evolution of fictive temperature subject to the forming temperature and deformation rate.

Using the modified model, following step-by-step definition of the 26 parameters and relationships, it was shown that the simulated equal-biaxial deformation behaviour compared qualitatively well to the experimental data at temperatures and strain rates analogous to the forming process. It was possible to predict the initial stiffness, yielding, necking, and anisotropic strain hardening stages of deformation over the wide temperature window and strain rate range. Errors concerning the simulated magnitude of yield stress, presented at temperatures of 135 and 140 °C at the reference strain rate and at temperatures at the highest strain rate of 16 s^−1^, is presumed to be as a result of the modelled limiting viscosity due to averaged gradients of Eyring plots. The proposed modified material model is seen to be in good overall quantitative agreement with the displacement-controlled biaxial data in principal directions, showing minimum and maximum errors of 4.74% and 14.2% respectively—insignificant when compared to the 36.1% error associated with the prediction of the original Buckley material model at a temperature of 130 °C and strain rate of 16 s^−1^, in the transverse direction.

Considering the applicability of the characterised model in replicating the biaxial deformation of PEEK films, as the first of its kind in literature, the use of the modified Buckley material model is promising for future implementation into finite element simulations for the prediction of material deformation during forming. In addition, not only is the modified Buckley material model solely applicable for the modelling of PEEK films but following appropriate characterisation, can provide a stable basis for the replication of the biaxial forming behaviour of any amorphous polymeric materials exhibiting post-yield anisotropic behaviour as a result of material fabrication or processing.

## Figures and Tables

**Table 1:** Specifications of planar biaxial stretching conditions [13].

**Temperature Range**	130–155 °C
**Strain Rate Range**	1–16 s^−1^
**Mode of Deformation**	Equal Biaxial (EB)

**Table 2:** Bond-stretching stresses and limiting viscosities for different temperatures at strain of 0.2 at 1 s−1, for EB deformation.

Temperature, *T* (°C)	Total True Stress, *σ* (MPa)	Conformational Stress, $\sigma^{c}$ (MPa)	Bond-Stretching Stress, $\sigma^{b}$ (MPa)	Limiting Viscosity, $\mu_{0}$ (MPa)
130	34.10	6.77	27.33	92.66
135	28.36	6.77	21.59	39.68
140	22.87	6.77	16.10	17.15
145	18.09	6.77	11.32	8.08
150	12.29	6.77	5.52	2.74
155	6.77	6.77	0	0

**Table 3:** Characterised constants for modified Buckley material model through biaxial testing methods.

	Property	Value	Unit
**Bond Stretching Part**	Shear Modulus, $G^{b}$	250	MPa
	Bulk Modulus, $K^{b}$	6250	MPa
	Shear Activation Volume, $V_{s}$	1.74 × 10^−3^	m^3^ mol^−1^
	Pressure Activation Volume, $V_{p}$	1.41 × 10^−4^	m^3^ mol^−1^
	Reference Viscosity, $\mu_{0}^{*}$	92.7	MPa
	Activation Enthalpy, $\Delta H_{0}$	301	kJ mol^−1^
	Limiting Temperature, $T_{\infty }$	465	K
	Viscosity Constant, $C^{v}$	399	K
	Initial Fictive Temp, $T_{f0}$	419	K
	Reference Fictive Temp, $T_{f}^{*}$	423	K
	$C_{1}$ − $T_{shifted}$	−0.0892	
	$C_{2}$ − $T_{shifted}$	5.90	
	Strain Induced Increase, $\Delta T_{f}$	$-0.253\;\times \;T_{shifted}$ + 109	K
	Rejuvenation Strain Range, $\varepsilon_{0}^{v}$	0.0058 × SR + 0.181	
	Fitting Parameter, r	1.60	
**Conformational Part**	μ	1.00	MPa
	$k_{1}$	2.16	MPa
	$k_{2}$	0.0150	
	ρ	0.585	
	β	4.02 × 10^−11^	⁰
	Reference Slippage Viscosity, $\gamma_{0}^{*}$	1.68 0.20	MPa (MD) MPa (TD)
	Limiting Temperature, $T_{\infty }$	5.94	K
	Viscosity Constant, $C^{v}$	3296	K
	$C_{1}$ − $\lambda_{crit}^{n}$	0.213	
	$C_{2}$ − $\lambda_{crit}^{n}$	6.47	
	Critical Network Stretch, $\lambda_{crit}^{n}$	$0.0128\;\times \;T_{shifted}$ − 4.30	K

**Table 4:** Comparison in observed root mean squared errors (%RMSE) between predicted and observed stress–strain behaviour, for the modified and original Buckley material model, at a temperature of 130 °C.

Model	Machine Direction		Transverse Direction	
	Strain Rate (s^−1^)		Strain Rate (s^−1^)	
	1	16	1	16
Original	5.874%	19.91%	21.42%	36.08%
Modified	5.294%	10.73%	11.87%	5.340%
